# Loss of salivary agglutinin induces changes in the salivary microbiome and accelerates development of oral cancer

**DOI:** 10.1186/s40168-026-02337-5

**Published:** 2026-04-10

**Authors:** Marcell Costa de Medeiros, Simon Fontaine, Erika Danella, Ethan Hillman, Thomas M. Schmidt, Allison Furgal, Deneen M. Wellik, Naohiro Inohara, Gabriel Núñez, Gen Li, Grace Y. Chen, Nisha J. D’Silva

**Affiliations:** 1 Periodontics and Oral Medicine, University of Michigan School of Dentistry, Ann Arbor, USA; 2 Department of Statistics, University of Michigan, 1011 North University Ave, Room G018, Ann Arbor, MI 48109-1078 USA; 3 Department of Bioinformatics, University of Michigan, Ann Arbor, USA; 4 Department of Microbiology and Immunology, University of Michigan, Ann Arbor, USA; 5 Department of Ecology & Evolutionary Biology, University of Michigan, Ann Arbor, USA; 6 Department of Pathology, University of Michigan, Ann Arbor, USA; 7 Department of Internal Medicine, University of Michigan Medical School, Ann Arbor, USA; 8 Department of Biostatistics, School of Public Health, University of Michigan, Ann Arbor, USA; 9 Rogel Cancer Center, University of Michigan, Ann Arbor, USA; 10 Department of Cell and Regenerative Biology, University of Wisconsin-Madison, Madison, WI USA; 11 Center for Infectious Disease Education and Research (CiDER), Osaka University, Suita, Osaka 565-0871 Japan

## Abstract

**Background:**

Salivary agglutinin, also known as deleted in malignant brain tumors 1 (DMBT1), is an anti-microbial protein. DMBT1 is low in saliva from patients with oral squamous cell carcinoma (OSCC) and dramatically increases after treatment, with accompanying microbial changes. While this suggests an association between DMBT1 suppression and changes in the oral microbiota, causation has not been established. DMBT1 is also a tumor suppressor protein; its loss promotes OSCC progression, but its role in OSCC development is unknown. In this study, OSCC development was investigated in a murine carcinogen model that simulates human OSCC. Microbiota were standardized between *Dmbt1* knockout (*Dmbt1*^*−/−*^) and wild-type (*Dmbt1*^+*/*+^) mice via interbreeding and co-housing. Saliva was collected at baseline and at 4, 8, 12, 16, and 22 weeks post-carcinogen initiation (stopped at 16 weeks). Tongues were harvested at week 22 for histopathology, and the salivary microbiome was profiled by 16S rRNA sequencing. Microbial diversity metrics and conditional dependence networks assessed community structure, while longitudinal patterns were analyzed using a locally sparse varying coefficient mixed model and functional principal component analysis (fPCA).

**Results:**

Despite microbiota standardization, *Dmbt1*^*−/−*^ and *Dmbt1*^+*/*+^ displayed differences in microbiome composition based on β-diversity metrics. At endpoint, carcinogen-treated *Dmbt1*^*−/−*^ showed higher OSCC prevalence and more aggressive invasion than *Dmbt1*^+*/*+^. Several OTUs, including those from Lachnospiraceae, *Sphingomonas*, Carnobacteriaceae, and Candidatus Saccharibacteria families, demonstrated differential abundance patterns over time, either genotype-specific, diagnosis-specific, or both. Notably, *Sphingomonas* and Lachnospiraceae exhibited time-dependent abundance differences in mice that developed OSCC. fPCA identified taxa with abundance trajectories that were different between OSCC and precancer and genotype specific.

**Conclusions:**

Thus, DMBT1 shapes salivary microbiota composition and protects against OSCC development. Dynamic, genotype-specific microbial shifts during carcinogenesis underscore the complex interplay between the oral microbiota and cancer progression.

Video Abstract

**Graphical Abstract:**

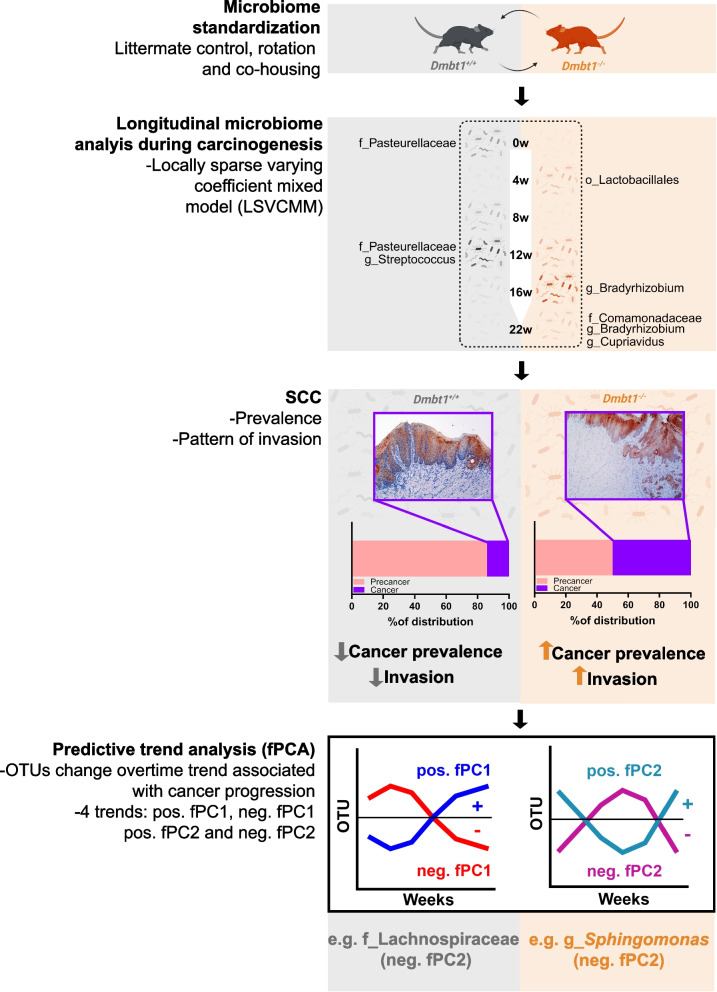

**Supplementary Information:**

The online version contains supplementary material available at 10.1186/s40168-026-02337-5.

## Introduction

The oral microbiota in the healthy human oral cavity has a diverse distribution mainly due to different habitats, including the hard surface of the tooth, soft tissue such as gingiva and tongue, and circulating saliva [1]. Squamous cell carcinomas of the oral cavity (OSCC) are continuously exposed to microbiota. Oral dysbiosis, a disruption in the balance of beneficial, commensal versus potentially harmful and pathogenic microbes, has been repeatedly associated with OSCC [2–8]. However, there is minimal understanding of the cause of dysbiosis and changes in microbial composition in precancer versus OSCC. In fact, association studies of dysbiosis and OSCC have been cross-sectional without establishing whether dysbiosis precedes or is secondary to OSCC [2, 4–7, 9]. Moreover, studies on dysbiosis and OSCC used different types of samples (tissue, saliva, etc.), which can vary in microbial populations [2, 4–7].

Mouthwash samples from OSCC and healthy controls found disease-related increases in *Fusobacterium periodonticum* and *Streptococcus constellatus* [10]. Comparisons of microbiota between the OSCC site and contralateral side showed a correlation between *Fusobacterium, Alloprevotella,* and *Porphyromonas* and tumor site [11]. A systematic review identified significant increases at the genus level of *Fusobacterium, Peptostreptococcus, Alloprevotella, Capnocytophaga, Catonella*, and *Prevotella* in patients with OSCC [12]. Based on these findings, a microbial dysbiosis index was proposed for use as a prognostic tool after appropriate validation [12].

Dysbiosis in saliva could also have treatment implications. For instance, we showed that responders and non-responders to chemoradiation for OSCC have significant differences in baseline microbiota [13]. There was a notable association between non-responders and the presence of *Prevotella* in saliva [13]. In contrast, responders exhibited a higher abundance of unclassified *Pasteurellaceae spp., Veillonella, Leptotrichia, Corynebacterium*, and *Lautropia*. In a separate study, *Bacteroidetes* was reduced in OSCC patients after radiotherapy to levels similar to that of healthy individuals, which is in contrast to pre-treatment, where the OSCC group had significantly higher levels [14].

Given the impact of the microbiota on progression and treatment resistance in other cancers [15], the salivary microbiome is likely important in modulating the development and progression of OSCC, which is continuously exposed to saliva. Understanding how oral microbial dysbiosis occurs and its role in OSCC could inform strategies to inhibit OSCC, including microbial modulation strategies to prevent development and progression.

Salivary agglutinin and other salivary glycoproteins maintain the commensal microbiota [16]. Also known as deleted in malignant brain tumors 1 (DMBT1), GP340, and SALSA, salivary agglutinin is a pattern recognition receptor that binds, aggregates, and eliminates bacteria in saliva by agglutination and swallowing [17–23]. Since DMBT1 constitutes up to ~10% of salivary protein [22], its loss is associated with dysbiosis [22, 24]. Not surprisingly, low DMBT1 in saliva or decreased binding to bacteria is associated with increased susceptibility to oral infection [22, 24]. For example, decreased binding of DMBT1 to *S. mutans* is associated with increased susceptibility to dental caries [23, 24].

DMBT1 is epigenetically silenced in OSCC [25] leading to loss of expression in epithelium [26]. We discovered that patients with OSCC have almost undetectable DMBT1 in saliva prior to treatment [13]; the dramatic post-treatment (6, 12 months) increase in DMBT1 is associated with significant shifts in microbial community structure [13]. These data are supportive, but not conclusive for loss of DMBT1 leading to oral dysbiosis and OSCC.

Surprisingly, though DMBT1 also regulates microbiota in the gut and lung [27, 28] and its expression is lost in multiple cancers [29], DMBT1-associated microbiome changes in any cancer are unknown. Given that the microbiota has an essential role in progression and treatment response in multiple cancers [30–32], understanding DMBT1’s anti-bacterial role would provide a strategy to promote eubiosis. In this in vivo study, we investigated the role of DMBT1 in regulating the composition of the oral microbiome and OSCC susceptibility. We demonstrate that loss of DMBT1 disrupts the salivary microbiota and accelerates the development of OSCC.

## Results

### DMBT1 deficiency promotes alterations in microbial composition and tumor development

**Fig. 1:**
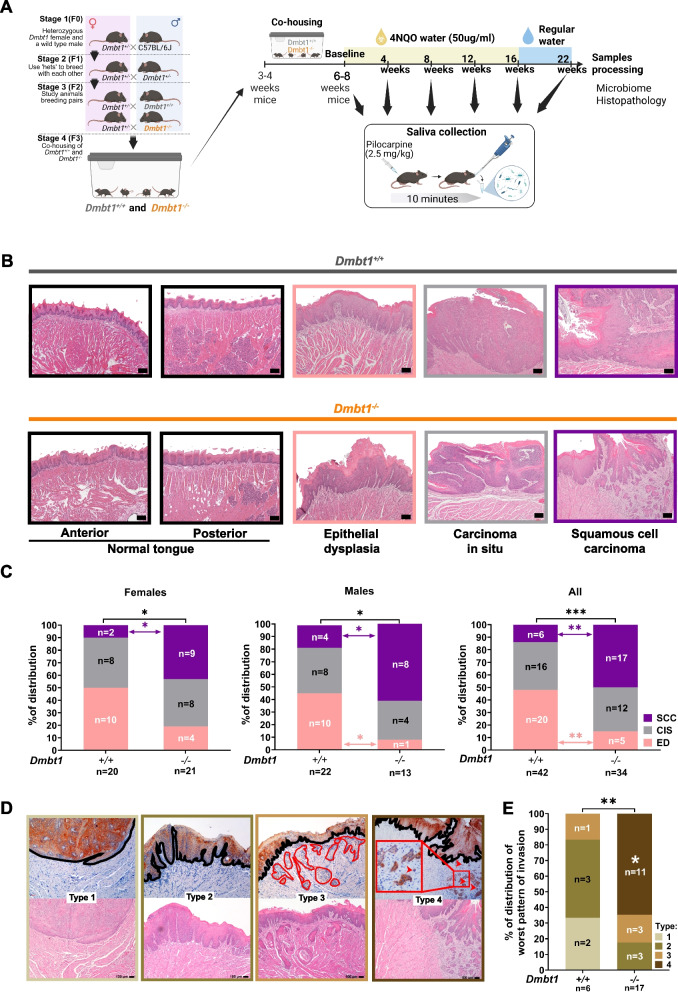
Loss of Dmbt1 promotes development of oral cancer. **A** Schematic of the breeding strategy used to standardize microbiota prior to 4NQO treatment. **B** H&E staining showing normal tongue epithelium, epithelial dysplasia, carcinoma-in-situ, and squamous cell carcinoma histopathology in *Dmbt1*^+*/*+^ and *Dmbt1*^*−/−*^ mice. Scale bars correspond to 100 µm, using a 20× magnification. **C** Distribution of precancers and OSCC in male and female *Dmbt1*^+*/*+^ and *Dmbt1*^*−/−*^. **D** Cytokeratin stain and H&E of tissue sections were used to score Types 1 through 4 patterns of invasion. Type 4 (small nests of tumor cells) is the most aggressive. Black lines delineate epithelium from stroma, and red lines and arrows point to satellites or tumor islands. Scale bars correspond to 100 µm, using a 20× magnification. **E** Distribution of each type of pattern of invasion in OSCC in *Dmbt1*^+*/*+^ and *Dmbt1*^*−/−*^. *p < 0.05, **p < 0.01, and ***p < 0.001

Our previous study in human saliva suggested that the loss of DMBT1 is associated with dysbiosis in OSCC [13], but a causal role was not established. In this study, we used *DMBT1*^*−/−*^ mice to address this knowledge gap. To control for colony-dependent effects on the gut microbiota, our breeding strategy included littermate controls and rotation of mice between cages to share microbiota and minimize microbial differences related to breeder ancestry independent of genotype [33] (Fig. 1A). Briefly, since maternal transmission influences the microbiota [34], heterozygous females carrying one allele of the *Dmbt1* gene (*Dmbt1*^±^) were crossed with wild-type males (*Dmbt1*^+*/*+^). From this breeding pair, six-week-old heterozygous *Dmbt1*^*−/*+^ progeny mice were used to establish a new breeding colony. Heterozygous females generated from these heterozygous crosses were then crossed with homozygous *Dmbt1*^*−/−*^ or *Dmbt1*^+*/*+^ male littermate. Their homozygous progeny was co-housed for 3–4 weeks after weaning, rotated between cages every other day, then used for carcinogen-mediated OSCC induction.

To induce OSCC, male (*Dmbt1*^+*/*+^ *n* = 22; *Dmbt1*^*−/−*^ *n* = 13) and female (*Dmbt1*^+*/*+^ *n* = 20; *Dmbt1*^*−/−*^ *n* = 21) mice were exposed to a carcinogen, 4-nitroquinoline-1-oxide (4NQO, 50 μg/mL in drinking water) for 16 weeks, followed by regular water for 6 weeks. 4NQO was selected because it induces mutations similar to those observed in human OSCC [35]. Saliva was collected at baseline, and 4, 8, 12, 16, and 22 weeks after carcinogen initiation to monitor microbial dynamics during carcinogenesis (Fig. 1A). At study endpoint, tongues were harvested; based on histopathology, lesions were classified as precancer (i.e., hyperplasia/epithelial dysplasia (ED)/carcinoma-in-situ (CIS), or cancer (OSCC)) (Fig. 1B).

Loss of DMBT1 increased susceptibility to OSCC (Fig. 1C) with greater numbers of *Dmbt1*^*−/−*^ than *Dmbt1*^+*/*+^ mice developing OSCC (*p* = 0.00525, Chi-square test). On the other hand, more *Dmbt1*^+*/*+^ mice exhibited epithelial dysplasia compared to *Dmbt1*^*−/−*^ mice (*p* = 0.00182, Chi-square test). There was no significant difference between the number of *Dmbt1*^+*/*+^ and *Dmbt1*^*−/−*^ that developed carcinoma-in-situ (*p* = 0.989, Chi-square test).

There were other differences between genotypes that were also gender specific. With females, more mice developed OSCC, but not carcinoma-in-situ, and there was a trend towards more epithelial dysplasia in *Dmbt1*^*−/−*^ than *Dmbt1*^+*/*+^ (OSCC: *p* = 0.0432, CIS: *p* = 1.000 and ED: *p* = 0.0784, Chi-square). In males, more *Dmbt1*^*−/−*^ mice developed OSCC, but not carcinoma-in-situ, than *Dmbt1*^+*/*+^ mice, whereas fewer *Dmbt1*^*−/−*^ mice had epithelial dysplasia compared to *Dmbt1*^+*/*+^ mice (ED: *p* = 0.0270, OSCC: *p* = 0.02442 and CIS: *p* = 1.00, Fisher’s exact test).

A histopathologic characteristic of OSCC that is associated with prognosis is the most aggressive pattern of invasion within the tumor (“worst pattern of invasion”). Specifically, high grade, i.e., small clusters of tumor cells at the invasive front, is associated with poor survival [36]. Therefore, we investigated the worst pattern of invasion in OSCC, which showed differences between genotypes (Fig. 1D, E). Based on hematoxylin and eosin (H&E) (Fig. 1B) and anti-cytokeratin (Fig. 1D) staining, and applying the worst-pattern-of-invasion score [37], OSCC in *Dmbt1*^*−/−*^ exhibited a more aggressive growth pattern than *Dmbt1*^+*/*+^ (Fig. 1E). Specifically, OSCC in *Dmbt1*^*−/−*^ mice exhibited nests of small invasive tumor islands (Type 4), whereas *Dmbt1*^+*/*+^ mainly exhibited finger-like projections, or large separate tumor islands (Types 1, 2, and 3) (Fig. 1E). Since fewer *Dmbt1*^+*/*+^ mice developed OSCC, the sample size was smaller than in the *Dmbt1*^*−/−*^ group. Altogether, these data show that *Dmbt1*^*−/−*^ are more susceptible to OSCC development than *Dmbt1*^+*/*+^ mice, and OSCC in *Dmbt1*^*−/−*^ exhibits an aggressive pattern of invasion. These results indicate a pivotal role for loss of DMBT1 in modulating the development and progression of oral cancer.

**Fig. 2:**
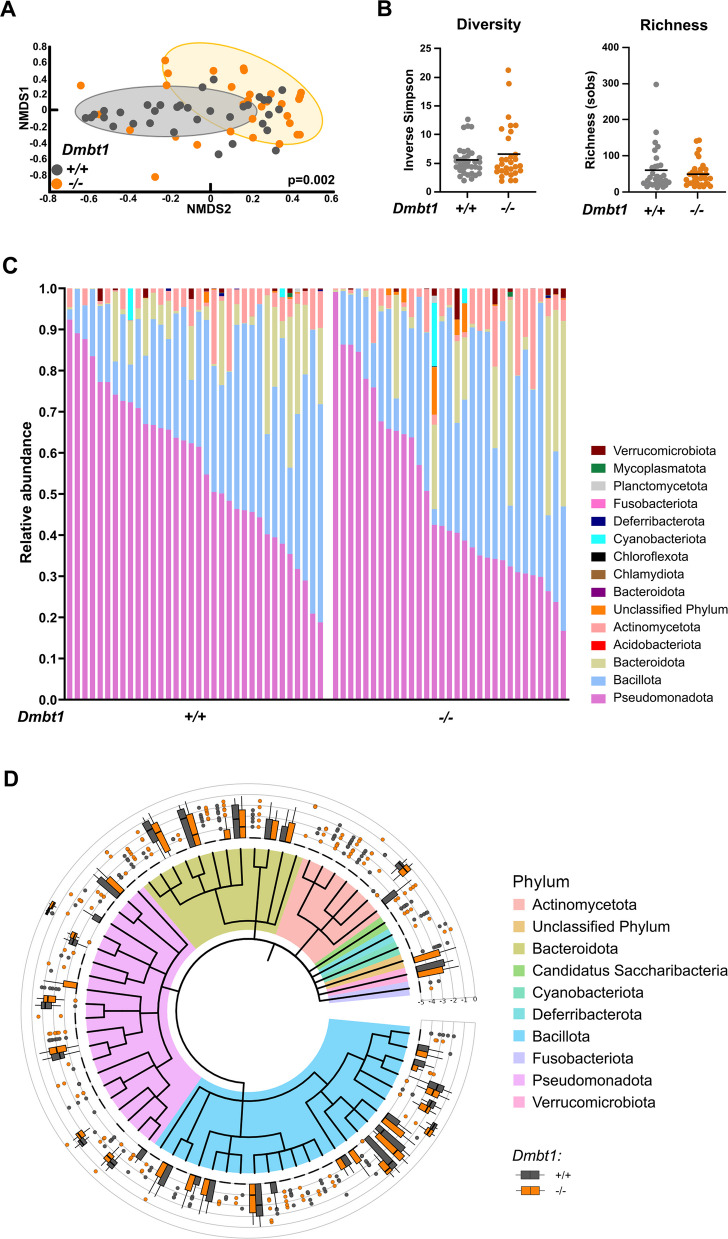
Baseline community dissimilarity between *Dmbt1*^+*/*+^ and *Dmbt1*^*−/−*^ mice at homeostasis. **A** Nonmetric multidimensional scaling (NMDS) ordination based on θ_YC_ distances between *Dmbt1*^+*/*+^ and *Dmbt1*^*−/−*^ microbiota at baseline prior to 4NQO treatment. Significance was determined using analysis of molecular variance (AMOVA). **B** Inverse Simpson’s diversity index and observed community richness. **C** Relative abundance community distribution at phylum level. **D** Histogram of log relative abundance aggregated at order level and organized by taxonomy and genotype

In a previous study, we showed an association between loss of salivary DMBT1 expression and microbial changes in OSCC patients [13]. Since DMBT1 is an anti-bacterial protein [22], we investigated the impact of loss of DMBT1 on the salivary microbiome in naïve, healthy mice to determine if DMBT1 is important for regulating the composition of the oral microbiome at homeostasis (Fig. 2). Despite inter-breeding and co-housing of mice to equilibrate microbiota, there were significant differences in oral microbial community structure between *Dmbt1*^+*/*+^ and *Dmbt1*^*−/−*^ at baseline based on dissimilarity of θ_YC_ distances (Fig. 2A; *p* = 0.002, AMOVA), suggesting that DMBT1 regulates the composition of the oral microbiota. However, the absence of DMBT1 did not affect overall diversity or richness (Fig. 2B). Moreover, at the phylum level, there were no significant differences in relative abundances of bacteria between *Dmbt1*^+*/*+^ and *Dmbt1*^*−/−*^ (Fig. 2C). Similarly, there were no significant differences in abundance of bacteria at the order level (Fig. 2D).

### Differential taxonomic interaction networks reveal genotype-specific patterns across timepoints during oral carcinogenesis

**Fig. 3:**
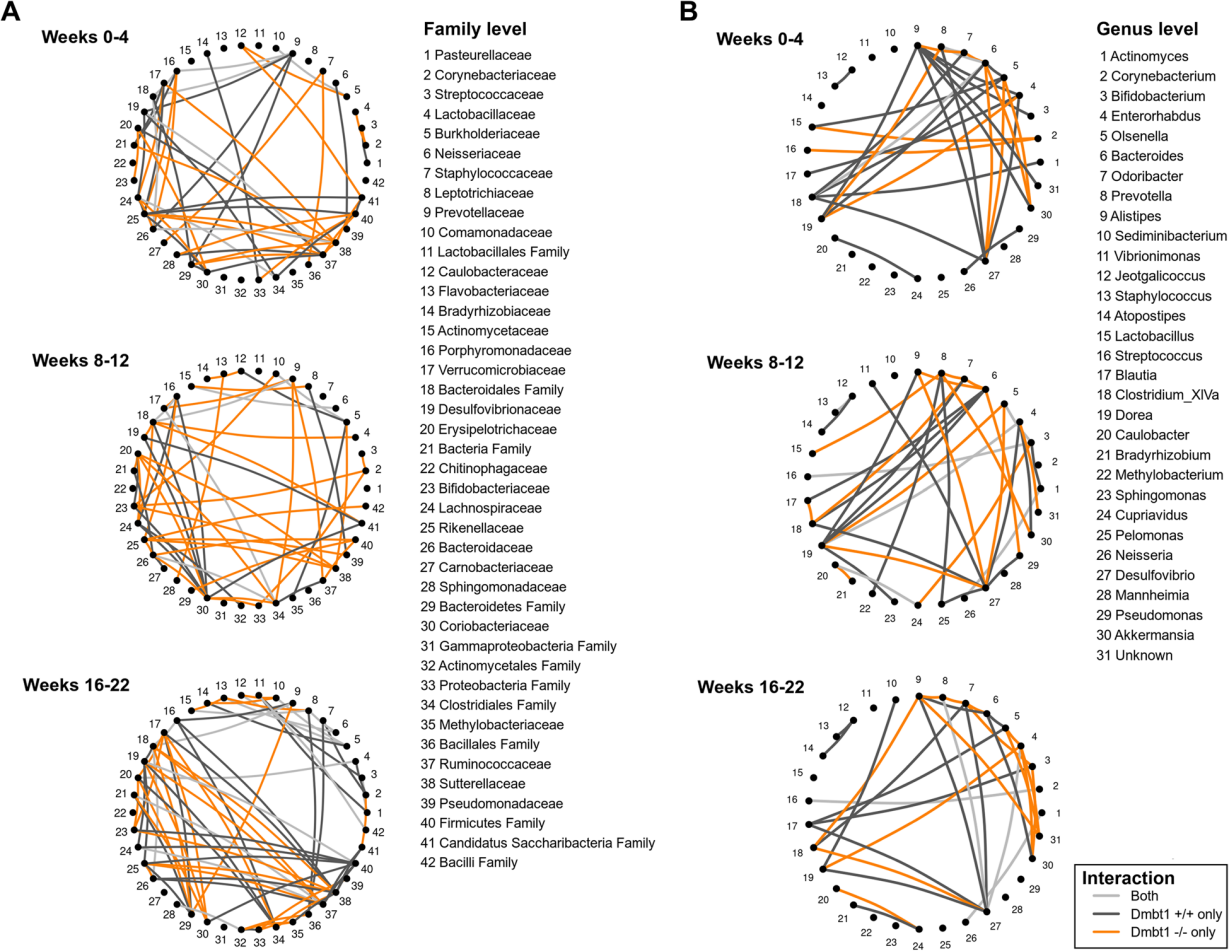
Covariations between taxa across time points and taxonomy levels differ between *Dmbt1*^+*/*+^ and *Dmbt1*^*−/−*^ mice. To analyze interactions between taxa across *Dmbt1* genotypes (*Dmbt1*^+*/*+^ and *Dmbt1*^*−/−*^), interaction taxonomic networks were constructed at family (**A**) and genus (**B**) taxonomic levels. The networks depict patterns of conditional dependence/independence among taxa, highlighting covariation relationships adjusted for confounder effects

To investigate relationships between taxa at the family and genus levels based on *Dmbt1* genotypes and how these interactions change with time during the development of precancer and OSCC, a network analysis was performed. There was covariation between taxa that were distinct between *Dmbt1*^+*/*+^ and *Dmbt1*^*−/−*^ at both family (Fig. 3A) and genus (Fig. 3B) levels, and there were very few interactions between taxa that were common to *Dmbt1*^+*/*+^ and *Dmbt1*^*−/−*^ mice at all timepoints, likely reflecting the dependence of interactions on genotype. One common interaction was between *Comamonadaceae* and *Burkholderiaceae* at all timepoints in both genotypes (Fig. 3A, nodes 10 and 5, respectively). Very few interactions remained constant with time, possibly due to the development of precancer and cancer after carcinogen exposure (Fig. 3A, B). A small core of interactions is stable across time and both genotypes, mainly involving Burkholderiaceae (5) and the Bacteroidales family (18), suggesting these two may be central nodes in the community (Fig. 3A). Many more interactions are stable only in *Dmbt1*^*⁻/⁻*^ mice, where Rikenellaceae (25) and Ruminococcaceae (37) repeatedly appear, indicating that these families are key drivers of the *Dmbt1*^*−/−*^-specific community structure (Fig. 3A). Far fewer interactions are specific to wild-type mice, and these do not show a clear recurring family pattern, suggesting that the *Dmbt1*^+*/*+^ network is less dominated by a small set of families compared with the *Dmbt1*^*⁻/⁻*^ network (Fig. 3A). Interestingly, at the genus level, there is an interaction between *Caulobacter* and *Cupriavidus* (Fig. 3B, nodes 20 and 24, respectively) that is prevalent in *Dmbt1*^+*/*+^ in weeks 0–4, in both *Dmbt1*^*−/−*^ and *Dmbt1*^+*/*+^ during weeks 8–12, and only in *Dmbt1*^*−/−*^ during weeks 16–22. Also observed was an interaction between *Enterorhabdus* and *Dorea* (Fig. 3B, nodes 4 and 19, respectively) that remained constant in *Dmbt1*^*−/−*^ at all time intervals and occurred in *Dmbt1*^+*/*+^ only in weeks 8–12. Overall, the network analysis highlights dynamic changes in microbiota during progression from normal epithelium to precancer and/or OSCC and that these changes are largely distinct between *Dmbt1*^+*/*+^ and *Dmbt1*^*−/−*^ mice, further supporting a role for DMBT1 in modulating oral microbiota composition that may be related to its effect on oral carcinogenesis.

### Differences in longitudinal changes in bacterial abundance based on genotype and diagnosis

**Fig. 4:**
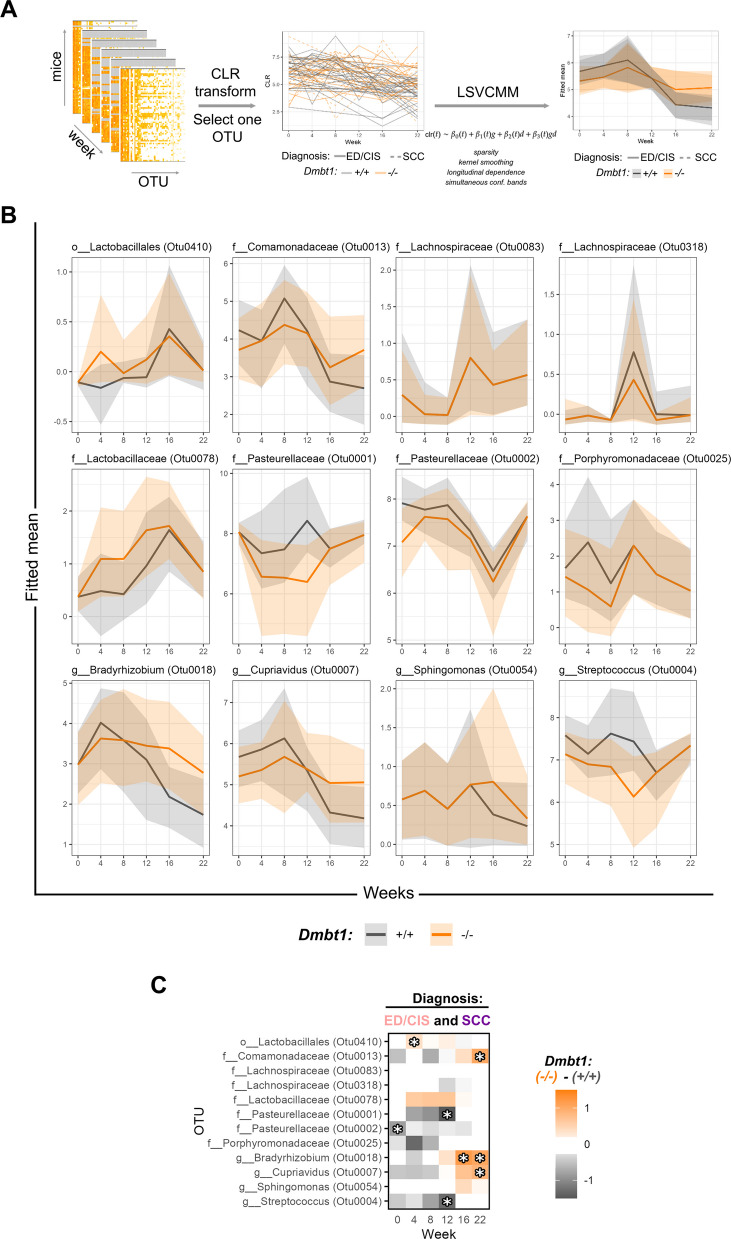
Significantly different OTUs between *Dmbt1*^+*/*+^ and *Dmbt1*^*−/−*^ mice at different timepoints and stratified by diagnosis. **A** Schematic of the Locally Sparse Varying Coefficient Mixed Model (LSVCMM) use for microbiome analysis. **B** Fitted mean (CLR-transformed) of the significant OTUs and changes with time based on genotype using the LSVCMM model. **C** Heatmap summarizing OTUs significantly different in abundance at any timepoint for ED/CIS and OSCC. Asterisks represent statistical significance

**Fig. 5:**
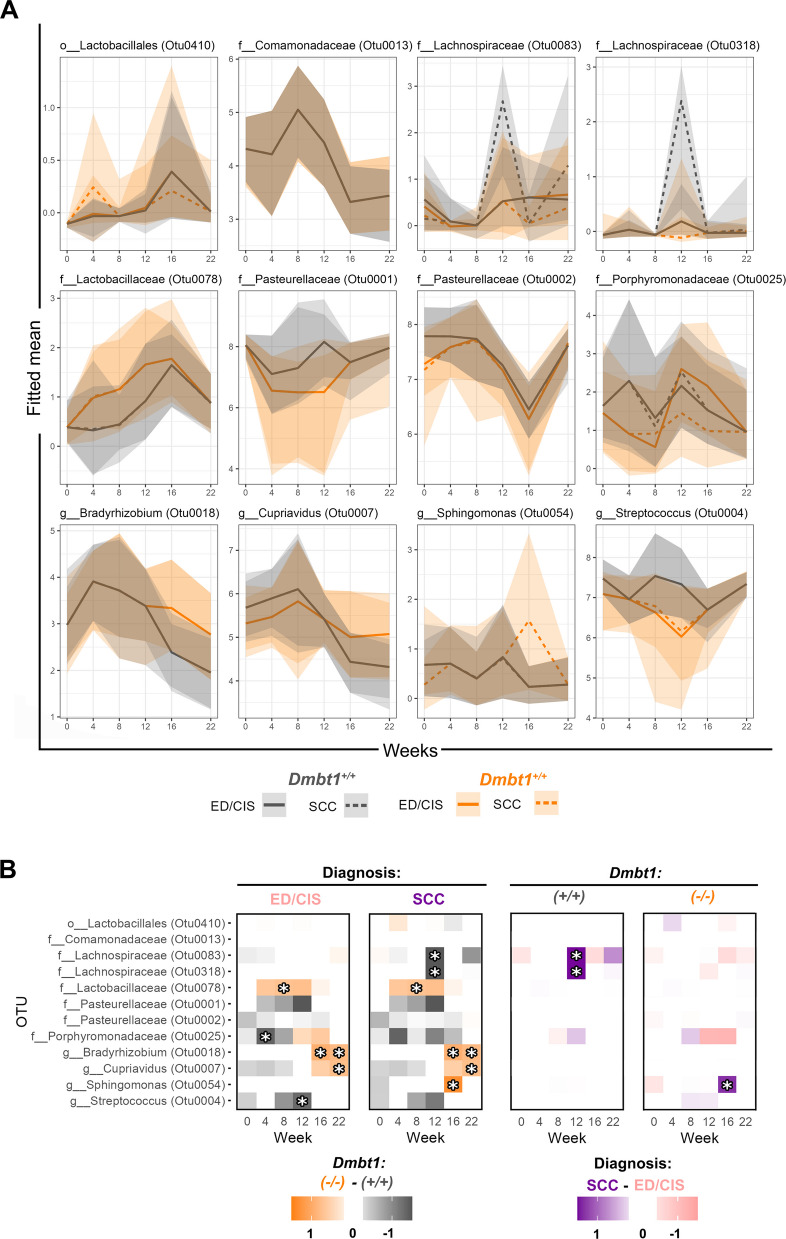
Distinct changes in abundance of OTUs associated with DMBT1 deficiency and disease state. **A** Fitted mean (CLR-transformed) of significant OTUs over time based on genotype and diagnosis. **B** Heatmap summarizing OTUs significantly different in abundance at any timepoint for ED/CIS, OSCC, *Dmbt1*^+*/*+^ and *Dmbt1*^*−/−*^. Asterisks represent statistical significance

To determine whether specific operational taxonomic units (OTUs), defined as bacteria with at least 97% sequence identity, differed between *Dmbt1*^+*/*+^ and *Dmbt1*^*−/−*^ and/or histopathology over time, a novel locally sparse varying coefficient mixed model (LSVCMM, Fontaine et al., manuscript under review at *Biometrics*), was developed (Fig. 4A). For this analysis, precancers, including hyperplasia/epithelial dysplasia and carcinoma in situ, were grouped together as one category (ED/CIS). After OTUs were Center Log-Ratio (CLR) transformed, we performed three analyses: (i) differences between genotypes, irrespective of diagnosis (Fig. 4B, C); (ii) differences between diagnosis, irrespective of genotype (Figure S1); and (iii) four pairwise differences (differences between genotypes in each diagnosis group and differences between diagnosis in each genotype) in the full model including interaction terms (Fig. 5). Figures 4B, S1A, and 5 A illustrate estimated mean abundance over time by genotype (*Dmbt1*^+*/*+^ versus *Dmbt1*^*−/−*^, Fig. 4B), diagnosis (ED/CIS versus OSCC, Figure S1), or their intersection (genotype and diagnosis) (Fig. 5A). Included are only those OTUs for which at least one pairwise comparison showed a significant difference for at least 1 week, established through a 95% simultaneous confidence band. The model including diagnosis only (Figure S1) did not identify any OTU that was differentially abundant between diagnoses at any week, which agrees with the interaction model (Fig. 5), where all significant differences between diagnoses occurred only within a single genotype.

There were 12 bacteria (OTUs) whose fitted CLR-transformed mean abundances were significantly different between genotypes or diagnoses at any timepoint, including multiple OTUs belonging to the family Lachnospiraceae and Pasteurellaceae (Fig. 5). Other OTUs included *Streptococcus* (OTU0004), an OTU belonging to Porphyromonodaceae (OTU0025), and *Sphingomonas* (OTU0054). Nine of these OTUs showed a similar pattern of increased abundance at various timepoints regardless of genotype, despite the level of significance sometimes changing between univariate and joint analyses (Figs. 4B, C, 5A, B). OTU0410 (order *Lactobacillales*) (Fig. 4B, C) and OTU0078 (*Lactobacillaceae*) (Fig. 5A, B) were more abundant in *Dmbt1*^*−*/−^ in weeks 4 and 8, respectively, after initiation of carcinogen. OTUs within the family *Comamonadaceae* (OTU0013) and within the genus *Bradyrhizobium* (OTU0018) and *Cupriavidus* (OTU0007) were found to be more abundant in *Dmbt1*^*−/−*^ mice at later timepoints, around weeks 16–22 (Figs. 4B, C, 5A, B). In *Dmbt1*^+*/*+^ mice, OTUs belonging to family *Pasteurellaceae* (OTU0002) and *Porphyromonadaceae* (OTU0025) were found to be more abundant at early timepoints (weeks 0–4). In contrast, OTU0001 belonging to *Pasteurellaceae* and OTU004 belonging to *Streptococcus* (OTU0004) were more abundant in *Dmbt1*^+*/*+^ mice around week 12 (Fig. 4B, C). The remaining three significantly different OTUs between genotypes showed differential trends that varied with diagnosis. Specifically, OTU0318 and OTU0083 that belong to Lachnospiraceae were found to be more abundant in *Dmbt1*^+/+^ mice around week 12, but only among mice that developed OSCC (Fig. 5A, B). OTU0054 belonging to *Sphingomonas* was more abundant in *Dmbt1*^*−/−*^ mice around week 16, but only among mice with OSCC (Fig. 5A, B). These three OTUs were also significantly different in abundance between mice that developed ED/CIS versus OSCC (Fig. 5). These differences were genotype-specific; Lachnospiraceae (OTU0318 and OTU0083) were more abundant around week 12 among mice diagnosed with OSCC, but only in *Dmbt1*^+*/*+^ mice, whereas *Sphingomonas* (OTU0054) was more abundant around week 16 among mice diagnosed with OSCC, but only in *Dmbt1*^*−/−*^ mice.

These findings suggest that loss of DMBT1 expression can influence which oral bacteria thrive and which may be associated with the development and progression of cancer. Notably, all differences in abundance between ED/CIS and OSCC are genotype-specific.

### Analysis of OTU abundance curves over time reveal genotype- and diagnosis-specific changes

**Fig. 6:**
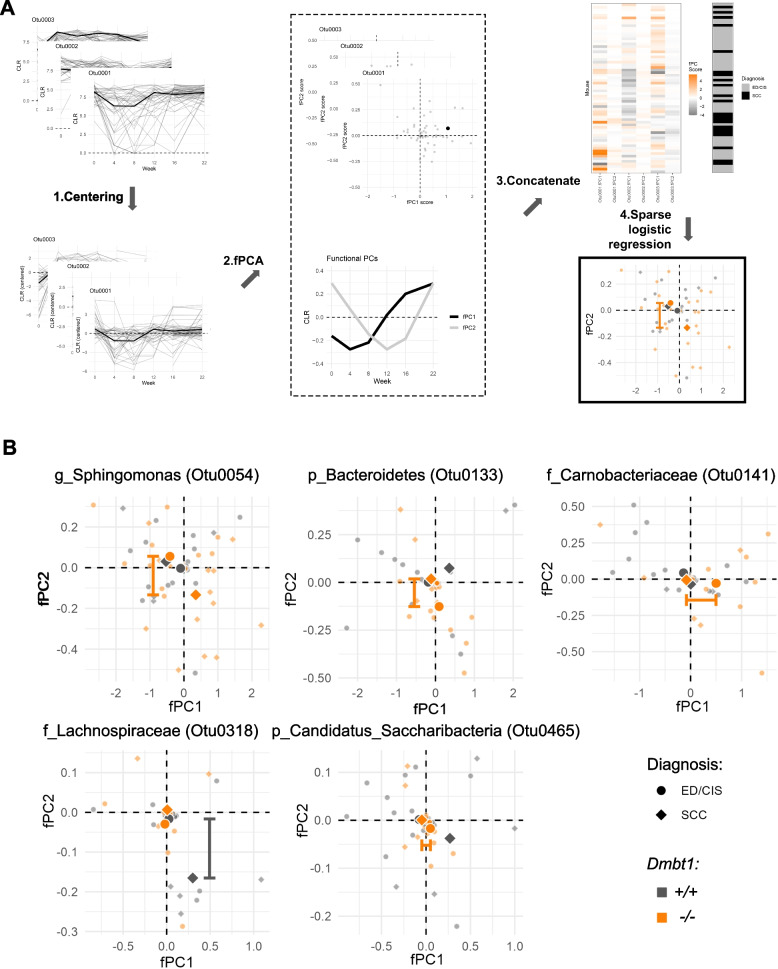
OTUs predictive of diagnosis in *Dmbt1*^+*/*+^ or *Dmbt1*^*−/−*^ mice. **A** Pattern of abundance changes over time as defined by functional principal component analysis (fPCA). Two principal components (fPCs) were identified in this analysis: fPC1 reflects increased abundance from week 8 to 16, while fPC2 indicates decreased abundance until week 12 followed by an increase from week 16. **B** Distribution of fPC scores and group average of OTUs in *Dmbt1*^+*/*+^ or *Dmbt1*^*−/−*^ identified to be predictive of diagnosis. fPCs that were predictive are represented by a bar between the corresponding group averages

Functional principal component analysis (fPCA) was performed to identify trends in OTU abundance over time that can be used to distinguish mice based on diagnosis and genotype. This type of analysis does not evaluate the absolute level of each OTU; rather, it evaluates if the temporal variability for each OTU can predict cancer status in either *Dmbt1*^+*/*+^ or *Dmbt1*^*−/−*^ mice and complements the LSVCMM analysis. For instance, LSVCMM estimates the expected abundance of a taxon given a genotype and precancer/cancer diagnosis whereas fPCA analysis informs whether the abundance trajectory of a specific OTU (e.g., increase over time or decrease then increase over time) can distinguish between diagnoses (Fig. 6) regardless of its absolute level. In this analysis, positive fPC1 values correspond to a pattern of change over time where the OTU abundance transitions from low to high between weeks 8 and 16, and positive fPC2 values correspond to a parabolic-like change with first a decrease in abundance until week 12, followed by an increase in abundance up to week 22 (Fig. 6A). Negative fPC1 and fPC2 values represent inverse trends compared to respective positive values (Fig. 6A). Only 5 OTUs (*i.e*., OTU0318, OTU0141, OTU0465, OTU0133, and OTU0054) had changes in abundance over time that were distinct between ED/CIS (circle) versus OSCC (diamond) and occurred mostly in *Dmbt1*^*−/−*^ mice with the exception of OTU0318, which occurred only in *Dmbt1*^+*/*+^ mice (Fig. 6B). More specifically, *Carnobacteriaceae* (OTU0141) and Candidatus *Saccharibacteria* (OTU0465) *Dmbt1*^*−/−*^ mice showed distinct abundance changes over time between ED/CIS (orange circle) and OSCC (orange diamond) with a positive group average fPC1 value, indicating that their abundance tends to increase starting around week 4 in *Dmbt1*^*−/−*^ mice that develop ED/CIS. *Sphingomonas* (OTU 0054) had a distribution of fPC scores that were distinct between ED/CIS and OSCC only among *Dmbt1*^*−/−*^ with a negative group average fPC2 value for mice with OSCC, indicating that the abundance of OTU0054 peaks around week 12 among *Dmbt1*^*−/−*^ mice (Fig. 6B). A different pattern was observed with a Bacteroidetes *spp.* (OTU0133) where its abundance profile clusters more closely to positive fPC2 values, indicating that its abundance tends to reach a minimum around week 12 only among *Dmbt1*^*−/−*^ mice (orange) that develop ED/CIS (circle) (Fig. 6B). Lachnospiraceae (OTU0318) is the only OTU that differentiates between ED/CIS and OSCC among *Dmbt1*^+*/*+^ mice (black). The distribution of fPC scores (Fig. 6B) indicates that *Dmbt1*^+*/*+^ that only developed ED/CIS (circle) did not have any common trend, while *Dmbt1*^+*/*+^ mice with OSCC had a negative fPC2 group average, indicating that the abundance of OTU0318 increases over time starting from week 0, peaks around week 12 before decreasing in abundance.

Overall, using this unique prediction model based on longitudinal microbiome changes that are associated with tumorigenesis and DMBT1 status, we identified taxa that may be a signature of precancer or cancer outcomes. These bacterial signatures will need validation.

## Methods

### Breeding strategy

Prior to initiation, all animal studies were approved by the University of Michigan Institutional Animal Care and Use Committee (IACUC). To eliminate microbiota differences due to ancestry and cage effects independent of genotype, we used a previously validated breeding strategy, including littermates and cohousing of mice [33]. As described in our previous study (PMID: 33835136), *Dmbt1*^*−/−*^ mice were generated by In vitro fertilization (IVF) using cryopreserved sperm from the *B6.129X1-Dmbt1tm1Kumc/Mmmh* (030289-MU-SPERM, MMRRC) on a C57BL/6 background (University of Michigan Transgenic Animal Model Core). Genotypes were confirmed per the supplier’s protocol for stock 30289 (PMID: 18202109). Initially, a heterozygous female (*Dmbt1*^±^*)* was bred with a wild type male C57BL/6 J (JAX #000664). F1 litters from this breeding pair, *Dmbt1*^±^ male and female, were bred to generate the F2 litter that was genotyped and bred as follows: (a) female *Dmbt1*^±^ and male *Dmbt1*^+*/*+^ to generate *Dmbt1*^+*/*+^; (b) female *Dmbt1*^±^ and male *Dmbt1*^*−/−*^ to generate *Dmbt1*^*−/−*^. The progeny, i.e., F3 *Dmbt1*^+*/*+^ and *Dmbt1*^*−/−*^ were co-housed for 3–4 weeks after weaning at 3 weeks, rotating every 2 days to permit microbiome exchange.

### Carcinogen model

The goal of the breeding strategy and cohousing was to standardize microbiota so that any differences identified were genotype related. To induce OSCC, the well-established 4-nitroquinoline-1-oxide (4NQO) carcinogen model was used [38, 39]. 6–8 week-old mice (age at baseline) received 4NQO (50 µg/ml; Millipore-Sigma, Cat N8141) in regular water for 16 weeks (changed weekly). Then, mice received regular water for an additional 6 weeks (tumor development window).

### Saliva collection

Saliva was collected at baseline (experimental week 0, prior to 4NQO), then 4, 8, 12, 16, and 22 weeks after 4NQO initial exposure with intraperitoneal injection of 2.5 mg/kg pilocarpine (0.5 µg/µl; i.e., 100 µl/20 20 g mouse). After 2 min, stimulated whole saliva was collected on ice by pipet for 10 min. Saliva was frozen immediately at −80 °C until processed for 16 s sequencing.

### Tissue processing, staining and scoring

After euthanasia, mouse tongues were isolated and fixed overnight with 4% paraformaldehyde, then sectioned perpendicular to the long axis, into 4 to 5 fragments, and embedded in paraffin. Tissue sections (4 µm) were stained with H&E for histopathology. Precancers included epithelial dysplasia/hyperplasia and carcinoma-in-situ [40].

Paraffin-embedded tissue sections (5 µm thick) were de-paraffinized in xylene, then incubated with 3% hydrogen peroxide in methanol to block endogenous peroxidase. For antigen retrieval, sections were boiled in citrate buffer (10 mM, pH 6.0, 15 min in microwave), then transferred to 1% BSA blocking buffer for 30 min. Primary antibody (33.3 ng/ml, Keratin 17/19, CST #12434) was incubated overnight, followed by biotin complexed secondary antibody (Biocare Medical). Slides were incubated with Streptavidin HRP Label (Biocare Medical), then 1 min with 3,3’ –diaminobenzidine (DAB) chromogen and counterstained with hematoxylin [41]. Control sections were incubated with IgG instead of primary antibody, at the same concentration.

Worst Pattern of Invasion [37] was scored based on H&E and cytokeratin stained slides that highlight tumor cells. Briefly, scores for the invasive pattern were: Type 1, tumor with pushing border; Type 2, finger-like tumor islands; Type 3, large, separate tumor islands with more than 15 cells; Type 4, small tumor islands with less than 15 cells (Fig. 1D).

### Microbiome sample preparation and DNA isolation/amplification

To isolate the bacterial pellet prior to submission to the Microbiome Core (University of Michigan), saliva samples were centrifuged at 10,000 rpm for 20 min at 4 °C. Supernatant was transferred to a new tube; the pellet was resuspended in 100 µl water and transferred to a DNA isolation plate (MagAtract PowerMicrobiome, Qiagen). The 16S rRNA gene V4 region was amplified and sequenced via Illumina MiSeq, as described [13].

### 16s microbiome sequencing data processing and analysis

Samples were stored at −80 °C until analysis. Microbial DNA was isolated with a MagAttract PowerMicrobiome DNA/RNA Kit (Qiagen) using an epMotion 5075 liquid handling system [42]. After extraction, amplicon samples were quantified using the Quant-iT PicoGreen dsDNA Assay (Invitrogen). Briefly, to analyze the microbial community composition, the V4 region of the 16S rRNA gene was amplified using established primers [43] and sequenced, as described [44] using the 2 × 250-bp paired end kits on the Illumina MiSeq Platform. For primary PCR, each 20μL PCR reaction was made up of the following: 5 μL equimolar (4 μM) primer set, 2 μL 10X AccuPrime PCR Buffer II (Life Technologies), 7.85 μL sterile PCR-grade water, 0.15 μL Accuprime High Fidelity Taq Polymerase (Life Technologies), and 5 μL template DNA. PCR cycling conditions were 95 °C for 2 min, then 30 cycles of standard PCR (95 °C for 20 s, 55 °C for 15 s, and 72 °C for 5 min), and finished with 72 °C for 10 min. PCR products were visualized using an E-Gel 96 with SYBR Safe DNA Gel Stain, 2% (Life Technologies). Amplicon libraries were normalized using SequalPrep Normalization Plate Kit (Life Technologies) following the manufacturer’s protocol for sequential elution. The concentration of the pooled samples was determined using a Kapa Biosystems Library Quantification kit for Illumina platforms. Amplicon sizes were determined using the Agilent Bioanalyzer High Sensitivity DNA analysis kit. Libraries were prepared according to Illumina’s “Preparing Libraries for Sequencing on the MiSeq” protocol for 2 nM libraries. The final library consisted of equimolar amounts from each of the plates normalized to the pooled plate at the lowest concentration. The final load concentration was 5.5 pM spiked with PhiX at 15% to add diversity. Sequencing reagents were prepared by the Michigan Microbiome Core as described [42]. Amplicons were sequenced using the Illumina MiSeq platform (San Diego, CA) using a MiSeq Reagent Kit V2 (Illumina) for 500 cycles. Sterile water (*n* = 4) and an empty DNA extraction control were sequenced as negative controls and a synthetic community (*n* = 4; ZymoBIOMICS Microbial Community DNA Standard) as positive controls.

The 16S rRNA sequence data were processed and analyzed using the mothur software package (v.1.45.3) and the most recent MiSeq analysis pipeline [45]. After sequence processing and alignment to the SILVA reference alignment (release 132), sequences were de-noised by collapsing sequences with 2 or fewer mismatches and then clustered into OTUs at the 97% level. Singletons and identified chimeric sequences were removed from the dataset. OTUs were classified using the RDP 16S rRNA gene database (version 18). Subsequently, sequences belonging to Chloroplast, Mitochondria, Archaea, Eukaryota, and unknown domains were removed. Each sample was rarefied to 9484 reads for downstream analysis [46]. Analysis of molecular variance (AMOVA) was performed to determine significance between the community structures of different groups based on θ_YC_ distance. Significant differences in microbial richness and diversity were assessed by Student’s *t*-test.

### Longitudinal differential interaction networks

We constructed conditional dependence networks at the family and genus levels using the SPRING method [47]; taxa with prevalence below 10% were discarded. The six time points were grouped into three periods: 0–4, 8–12, and 16–22 weeks. The StARS approach [48] was used for tuning parameter selection. The analysis was repeated for *Dmbt1*^*−/−*^ and *Dmbt1*^+*/*+^ samples.

### Experimental models developed to fit the longitudinal aspect of the study (LSVCMM)

To identify OTUs with differential abundance between genotype and diagnosis at any timepoint, we applied the methodology of Fontaine et al. (http://arxiv.org/abs/2601.10872) consisting of a locally sparse varying coefficient mixed model (LSVCMM). Specifically, we transformed abundances using the centered log-ratio transformation [49] where zeros were replaced by half the smallest nonzero count within each sample. We filtered out OTUs with prevalence below 5%, leaving 187 OTUs. The specific varying coefficient model consists of the regression of each OTU against five variables with time-varying coefficients: an intercept, an indicator for *Dmbt1*^*−/−*^, an indicator for OSCC, the interaction term *Dmbt1*^*−/−*^: OSCC, and an indicator for Female sex. Additionally, two simpler models were considered: one with an intercept, an indicator for *Dmbt1*^*−/−*^*,* and an indicator for Female sex; one with an intercept, an indicator for OSCC, and an indicator for Female sex. A compound symmetry covariance structure is used to account for longitudinal effects. Tuning parameter selection is done using the extended Bayesian information criterion proposed in Fontaine et al. (2024). We obtain bootstrap simultaneous 95% confidence bands for the following four contrasts: the difference between genotypes for each of the two diagnosis groups and the difference between diagnosis for each of the two genotypes.

### Predictive trend analysis

The longitudinal differential abundance analysis identifies mean differences in abundance between genotypes and diagnoses at specific weeks. In this analysis, we investigate whether overall temporal trends in abundance can discriminate between diagnoses. For each sample and for each OTU, we compute the CLR-transformed abundance trajectory as a six-dimensional vector, possibly with missing entries. Then, each trajectory is centered to remove mean differences. A fPCA [50] method is used to extract the most common trends among all trajectories (the functional PCs, fPC); trajectories are then summarized by the fPC scores. The number of components is selected to account for 99% of the variance. Then, the fPC scores are used as features in two Lasso Logistic regression models [51] predicting diagnosis, one for each genotype. The regularization parameter is selected using cross-validation.

## Statistics

### Diagnosis and worst pattern of invasion

To evaluate differences in ED/CIS and OSCC while controlling for sex, the Cochran-Mantel–Haenszel test was utilized. To compare statistical differences in all outcomes between *Dmbt1*^+*/*+^ and *Dmbt1*^*−/−*^ across all data, the Chi-square test was used. When examining the differences in diagnosis within each gender, the Chi-square test was applied for females, and Fisher’s exact test was used for males, respectively. For the worst pattern of invasion, the Fisher’s exact test was used.

## Discussion

DMBT1 is an anti-microbial protein in saliva and a tumor suppressor in oral epithelium. In the current longitudinal study, using *Dmbt1*^+*/*+^ and *Dmbt1*^*−/−*^ mice, we establish that DMBT1 regulates salivary microbial composition at homeostasis. Moreover, DMBT1 expression in oral epithelium protects against OSCC, which arises from the oral epithelium. A predictive model facilitated the identification of specific OTUs that were differentially abundant between genotypes and disease states, revealing unique microbial signatures associated with DMBT1 status and tumor progression. Dynamic longitudinal changes in microbiota due to DMBT1 loss and carcinogenesis suggest a complex relationship between the oral microbiota and cancer.

OSCC is associated with oral dysbiosis [52], an imbalance in the oral microbiome due to poor oral health [9, 53, 54]. Despite multiple attempts to associate specific bacteria and community compositions with OSCC to determine causality, most of these cross-sectional studies compared microbiota between patients with OSCC versus healthy controls. An increase in *Fusobacterium, Prevotella*, and *Gemella* species and a decrease in *Streptococcus* and *Rothia* species were reported in OSCC [55, 56]. The dynamic nature of microbial community structure over time suggests that short-term or cross-sectional studies may overlook important temporal fluctuations [57]. Similar to studies that reported subject-specific or condition-driven microbial dynamics [58, 59], our data suggest that certain microbial taxa follow unique, non-linear abundance patterns that may be critical to understand ecological stability, resilience, or disease progression. Our data demonstrate that OTUs can show transient increases or declines in abundance that correlate with key environmental or host-associated events, emphasizing the importance of longitudinal designs in microbiome research [60]. In addition, bacterial networks that develop during carcinogenesis may also be important. A nuanced understanding of bacterial-bacterial and microbiome-host interactions can facilitate the identification of time-sensitive biomarkers in systems which are tightly regulated.

Our previous longitudinal study showed for the first time that patients with OSCC had almost undetectable levels of salivary DMBT1 prior to treatment, followed by a dramatic increase in expression after treatment with accompanying microbial changes [13]. While this suggested an association between DMBT1 suppression and microbial changes, causation was not established. In this study, we investigated longitudinal microbial changes in a murine carcinogen model that simulates human OSCC [38, 39].

Suppression of DMBT1 in saliva has been associated with microbial changes [13, 26] but causality has not been established. After stringent breeding and co-housing of *Dmbt1*^*−/−*^ and *Dmbt1*^+*/*+^, the salivary microbiome was profiled at baseline, 4, 8, 12, 16, and 22 weeks after the initiation of carcinogen, which was stopped at 16 weeks. Based on β-diversity, the overall community structure between the salivary microbiome of *Dmbt1*^*−/−*^ and *Dmbt1*^+*/*+^ mice were significantly different at baseline. Moreover, longitudinal analysis revealed distinct changes in abundance trajectories for several individual OTUs, many previously undescribed, between *Dmbt1*^*−/−*^ and *Dmbt1*^+*/*+^ mice. For example, OTUs belonging to Lachnospiraceae and *Sphingomonas* were differentially abundant between *Dmbt1*^*−/−*^ and *Dmbt1*^+*/*^ mice at specific timepoints and also between OSCC and ED/CIS. In *Dmbt1*^*−/−*^ mice, *Carnobacteriaceae* (OTU0141) and Candidatus *Saccharibacteria* (OTU0465) increased over time in mice with precancer, while *Sphingomonas* (OTU0054) and *Bacteroidetes* (OTU0133) peaked around week 12 in mice with either OSCC or precancer, respectively. Lachnospiraceae (OTU0318) distinguished OSCC from precancer diagnoses only in *Dmbt1*^+*/*+^ mice. Together, these findings indicate that loss of salivary DMBT1 is linked to dysbiosis that both precedes the onset of ED/CIS and evolves with progression to OSCC.

Notably, the identification of OTUs belonging to *Sphingomonas* (OTU0054) and Lachnospiraceae (OTU0318) as differentially abundant between *Dmbt1*^*−/−*^ and *Dmbt1*^+*/*+^ and between precancer and OSCC diagnoses by both LSVCMM and fPCA analyses suggests that these OTUs may be significant for OSCC development and possibly regulated in some way by DMBT1. *Sphingomonas* OTU0054 exhibited significant abundance changes over time in *Dmbt1*^*−/−*^, but not *Dmbt1*^+*/*+^ mice. The *Sphingomonas* genus has been reported to be increased in gastric cancer compared to non-tumor tissues [61]. Similarly, in lung cancer, *Sphingomonas* was more prevalent in the tumor microbiome [62]. Additionally, in female non-smokers who developed lung cancer, the salivary microbiome revealed elevated levels of *Sphingomonas* compared to healthy oral microbiomes [63]. Interestingly, studies have described the presence of intratumoral *Sphingomonas* and increased prevalence of *Sphingomonas* in the deep tissues of OSCC compared to normal tissue [64]. The presence of this genus in the tumor microbiome was associated with better prognosis [65].

Two OTUs belonging to Lachnospiraceae family (OTU00318 and OTU0083) were enriched in *Dmbt1*^+*/*+^ that developed OSCC compared to *Dmbt1*^*−/−*^ (Fig. 5) and OTU00318 showed abundance changes over time that were also predictive of diagnosis in *Dmbt1*^+*/*+^ mice (Fig. 6) Lachnospiraceae showed increased levels in saliva and tongue microbiota of breast cancer patients compared to normal tissue [66]. Additionally, specific Lachnospiraceae species have been associated with OSCC, particularly those preceded by proliferative verrucous leukoplakia [67]. These findings highlight *Sphingomonas* and Lachnospiraceae as potentially linked to OSCC development, with patterns influenced by DMBT1. Their identification by both LSVCMM and fPCA supports their relevance, and previous associations with multiple cancers support their significance. Moreover, regulation of *Sphingomonas* by DMBT1 points to a possible host–microbe interaction that merits further investigation.

While loss of DMBT1 promotes OSCC progression [26], the effect on OSCC development has not been investigated. The present study established a role for DMBT1 in promoting OSCC development. Loss of DMBT1 significantly increases cancer susceptibility, with OSCC appearing earlier in *Dmbt1*^*−/−*^ mice compared to their wild type littermates. Moreover, OSCC in *Dmbt1*^*−/−*^ mice demonstrated more aggressive invasion than wild type mice. These results are consistent with studies that uncovered a tumor suppressor role for DMBT1; its loss in OSCC leads to tumor progression, including spread [26]. In the current study, it is unclear if loss of DMBT1 leads to rapid initiation of epithelial dysplasia or rapid progression of precancer to OSCC. This sequence could be directly established by temporal tissue collection.

Multiple epidemiologic studies have identified bacteria that are associated with cancer development, progression, and response to treatment, suggesting that the microbiome can present diagnostic and prognostic biomarkers [68]. While this has led to the inclusion of polymorphic microbiomes as a hallmark of cancer, much of this influence on cancer development and progression has focused on gastric and colorectal cancer [69]. The involvement of microbiota in carcinogenesis at other sites in the gastrointestinal tract, such as the oral cavity, remains understudied [70]. Due to a notable lack of longitudinal studies examining changes in the oral microbiota with OSCC development, progression, or treatment response, identification of specific microbial biomarkers has been a challenge [9, 71].

Our findings highlight the pivotal role of DMBT1 in modulating the salivary microbiota and cancer development and suggest a potential mechanism for dysbiosis in OSCC. DMBT1 accounts for up to 10% of salivary protein [22–24] and was first identified as a 300–400 kDa salivary streptococcal agglutinating protein [72]; hence the alternate name, salivary agglutinin. Subsequently, DMBT1 was identified in multiple tissues including brain and lung [73, 74]. DMBT1 protein has 13 scavenger receptor cysteine-rich (SRCR) domains connected by proline-rich segments, designated SRCR interspersed domains (SIDs). The 14th SRCR domain is flanked by two complement C1r/C1s, urokinase-type plasminogen activator, and bone morphogenetic protein 1 (CUB) domains followed by a zona pellucida (ZP) domain at the C-terminus [75, 76]. Bacterial binding properties of DMBT1 are associated with SRCR domains and may be a potential mechanism of its regulation of oral microbial composition. SRCRP2, a peptide sequence in the SRCR domain, has a high affinity for *S. mutans *[77, 78] and a broad range of bacterial species, including *S. gordonii, S. sanguis, S. oralis, S. sobrinus, S. mitis I, S. mitis II, P. intermedia, E. coli, A. actinomycetemcomitans, B. fragilis, M. catarrhalis, P. micros, S. aureus, L. casei*, and *H. pylori* [78, 79]. The length of the SRCR domain has also been implicated in binding to bacteria [19]. Besides SRCR domains, DMBT1 has anti-bacterial defense mechanisms including N-glycosylation, which inhibits the twitching motility responsible for the virulence of *Pseudomonas aeruginosa *[19], by binding to its pili [19, 80]. Low expression or absence of DMBT1 in saliva or decreased binding to bacteria is associated with susceptibility to oral infection [22–24]. For example, decreased binding of DMBT1 to *S. mutans* is associated with susceptibility to dental caries [23, 24]. Whether its bacterial binding properties relate to its ability to affect oral microbiome composition and susceptibility to carcinogenesis remains to be determined.

In oral inflammatory disease, bacteria disrupt epithelial integrity to release cytokines, some of which are also pro-tumorigenic and promote tissue invasion [81]. For example, co-culture of bacteria with keratinocytes enhances the release of matrix metalloproteinases (MMP) that promote invasion in cancer [82–86]. Of interest, loss of DMBT1 also decreases epithelial integrity and increases MMP9 [26]; in the current study, we show that loss of DMBT1 results in alterations in the salivary microbiome and accelerates the development of OSCC. It is tempting to speculate that DMBT1-induced changes in microbiota promote OSCC development. This could be addressed by microbial transfer to germ-free mice followed by carcinogen treatment. However, since the loss of DMBT1 alters the oral microbiota, transfer to germ-free mice that are not DMBT1-deficient may not provide the appropriate environment for bacterial differences to persist.

In conclusion, the current study establishes that loss of DMBT1 accelerates the development of OSCC in a robust pre-clinical model. We also establish that loss of DMBT1 in saliva induces microbial changes that precede the development of precancer and OSCC and is associated with complex alterations in microbiome composition during neoplastic development.

## Supplementary Information

Supplementary Material 1.

## Data Availability

The sequence data generated in this study has been deposited in NCBI under BioProject ID PRJNA1309046.

## References

[1] Dewhirst FE, Chen T, Izard J, Paster BJ, Tanner AC, Yu WH, et al. The human oral microbiome. J Bacteriol. 2010;192(19):5002–17. 10.1128/JB.00542-10. 20656903 10.1128/JB.00542-10 PMC2944498

[2] Burcher KM, Burcher JT, Inscore L, Bloomer CH, Furdui CM, Porosnicu M. A review of the role of oral microbiome in the development, detection, and management of head and neck squamous cell cancers. Cancers (Basel). 2022. 10.3390/cancers14174116. 36077651 10.3390/cancers14174116 PMC9454796

[3] Frank DN, Qiu Y, Cao Y, Zhang S, Lu L, Kofonow JM, et al. A dysbiotic microbiome promotes head and neck squamous cell carcinoma. Oncogene. 2022;41(9):1269–80. 10.1038/s41388-021-02137-1. 35087236 10.1038/s41388-021-02137-1 PMC8882136

[4] Guerrero-Preston R, White JR, Godoy-Vitorino F, Rodriguez-Hilario A, Navarro K, Gonzalez H, et al. High-resolution microbiome profiling uncovers *Fusobacterium nucleatum*, *Lactobacillus gasseri*/*johnsonii*, and *Lactobacillus vaginalis* associated to oral and oropharyngeal cancer in saliva from HPV positive and HPV negative patients treated with surgery and chemo-radiation. Oncotarget. 2017;8(67):110931–48. 10.18632/oncotarget.20677. 29340028 10.18632/oncotarget.20677 PMC5762296

[5] Lim Y, Fukuma N, Totsika M, Kenny L, Morrison M, Punyadeera C. The performance of an oral microbiome biomarker panel in predicting oral cavity and oropharyngeal cancers. Front Cell Infect Microbiol. 2018;8:267. 10.3389/fcimb.2018.00267. 30123780 10.3389/fcimb.2018.00267 PMC6085444

[6] Liu Y, Li Z, Qi Y, Wen X, Zhang L. Metagenomic analysis reveals a changing microbiome associated with the depth of invasion of oral squamous cell carcinoma. Front Microbiol. 2022;13:795777. 10.3389/fmicb.2022.795777. 35222330 10.3389/fmicb.2022.795777 PMC8863607

[7] Mager DL, Haffajee AD, Devlin PM, Norris CM, Posner MR, Goodson JM. The salivary microbiota as a diagnostic indicator of oral cancer: a descriptive, non-randomized study of cancer-free and oral squamous cell carcinoma subjects. J Transl Med. 2005;3(1):27. 10.1186/1479-5876-3-27. 15987522 10.1186/1479-5876-3-27 PMC1226180

[8] Stashenko P, Yost S, Choi Y, Danciu T, Chen T, Yoganathan S, et al. The oral mouse microbiome promotes tumorigenesis in oral squamous cell carcinoma. mSystems. 2019. 10.1128/mSystems.00323-19. 31387932 10.1128/mSystems.00323-19 PMC6687944

[9] Healy CM, Moran GP. The microbiome and oral cancer: more questions than answers. Oral Oncol. 2019;89:30–3. 10.1016/j.oraloncology.2018.12.003. 30732955 10.1016/j.oraloncology.2018.12.003

[10] Yang CY, Yeh YM, Yu HY, Chin CY, Hsu CW, Liu H, et al. Oral Microbiota Community Dynamics Associated With Oral Squamous Cell Carcinoma Staging. Front Microbiol. 2018;9:862. 10.3389/fmicb.2018.00862. 29774014 10.3389/fmicb.2018.00862 PMC5943489

[11] Zhang L, Liu Y, Zheng HJ, Zhang CP. The oral microbiota may have influence on oral cancer. Front Cell Infect Microbiol. 2019;9:476. 10.3389/fcimb.2019.00476. 32010645 10.3389/fcimb.2019.00476 PMC6974454

[12] Ting HSL, Chen Z, Chan JYK. Systematic review on oral microbial dysbiosis and its clinical associations with head and neck squamous cell carcinoma. Head Neck. 2023;45(8):2120–35. 10.1002/hed.27422. 37249085 10.1002/hed.27422

[13] Medeiros MC, The S, Bellile E, Russo N, Schmitd L, Danella E, et al. Salivary microbiome changes distinguish response to chemoradiotherapy in patients with oral cancer. Microbiome. 2023;11(1):268. 10.1186/s40168-023-01677-w. 38037123 10.1186/s40168-023-01677-w PMC10687843

[14] de Freitas Neiva Lessa A, da Silva Amancio AMT, de Oliveira ACR, de Sousa SF, Caldeira PC, De Aguiar MCF, et al. Assessing the oral microbiome of head and neck cancer patients before and during radiotherapy. Support Care Cancer. 2024;32(11):752. 10.1007/s00520-024-08953-x. 39470839 10.1007/s00520-024-08953-x

[15] Buchta Rosean C, Feng TY, Azar FN, Rutkowski MR. Impact of the microbiome on cancer progression and response to anti-cancer therapies. Adv Cancer Res. 2019;143:255–94. 10.1016/bs.acr.2019.03.005. 31202360 10.1016/bs.acr.2019.03.005 PMC7821737

[16] Cross BW, Ruhl S. Glycan recognition at the saliva - oral microbiome interface. Cell Immunol. 2018;333:19–33. 10.1016/j.cellimm.2018.08.008. 30274839 10.1016/j.cellimm.2018.08.008 PMC6296888

[17] Bathum Nexoe A, Pedersen AA, von Huth S, Detlefsen S, Hansen PL, Holmskov U. Immunohistochemical localization of Deleted in Malignant Brain Tumors 1 in Normal Human Tissues. J Histochem Cytochem. 2020;68(6):377–87. 10.1369/0022155420927109. 32436776 10.1369/0022155420927109

[18] Bikker FJ, End C, Ligtenberg AJM, Blaich S, Lyer S, Renner M, et al. The scavenging capacity of DMBT1 is impaired by germline deletions. Immunogenetics. 2017;69(6):401–7. 10.1007/s00251-017-0982-x. 28364129 10.1007/s00251-017-0982-x PMC5435793

[19] Li J, Wan SJ, Metruccio MME, Ma S, Nazmi K, Bikker FJ, et al. DMBT1 inhibition of *Pseudomonas aeruginosa* twitching motility involves its N-glycosylation and cannot be conferred by the Scavenger Receptor Cysteine-Rich bacteria-binding peptide domain. Sci Rep. 2019;9(1):13146. 10.1038/s41598-019-49543-w. 31511582 10.1038/s41598-019-49543-w PMC6739395

[20] Malamud D, Abrams WR, Barber CA, Weissman D, Rehtanz M, Golub E. Antiviral activities in human saliva. Adv Dent Res. 2011;23(1):34–7. 10.1177/0022034511399282. 21441478 10.1177/0022034511399282 PMC3144043

[21] Patyka M, Malamud D, Weissman D, Abrams WR, Kurago Z. Periluminal distribution of HIV-binding target cells and Gp340 in the oral, cervical and sigmoid/rectal mucosae: a mapping study. PLoS One. 2015;10(7):e0132942. 10.1371/journal.pone.0132942. 26172445 10.1371/journal.pone.0132942 PMC4501766

[22] Reichhardt MP, Holmskov U, Meri S. SALSA-a dance on a slippery floor with changing partners. Mol Immunol. 2017;89:100–10. 10.1016/j.molimm.2017.05.029. 28668353 10.1016/j.molimm.2017.05.029

[23] Zhang S, Huo X, Zhang Y, Lu X, Xu C, Xu X. The association of PM2.5 with airway innate antimicrobial activities of salivary agglutinin and surfactant protein D. Chemosphere. 2019;226:915–23. 10.1016/j.chemosphere.2019.04.032. 31509921 10.1016/j.chemosphere.2019.04.032

[24] Esberg A, Sheng N, Marell L, Claesson R, Persson K, Boren T, et al. *Streptococcus mutans* adhesin biotypes that match and predict individual caries development. EBioMedicine. 2017;24:205–15. 10.1016/j.ebiom.2017.09.027. 28958656 10.1016/j.ebiom.2017.09.027 PMC5652290

[25] Imai MA, Moriya T, Imai FL, Shiiba M, Bukawa H, Yokoe H, et al. Down-regulation of DMBT1 gene expression in human oral squamous cell carcinoma. Int J Mol Med. 2005;15(4):585–9 (PubMed PMID: 15754018). 15754018

[26] Singh P, Banerjee R, Piao S, Costa de Medeiros M, Bellile E, Liu M, Damodaran Puthiya Veettil D, Schmitd LB, Russo N, Danella E, Inglehart RC, Pineault KM, Wellik DM, Wolf G, D'Silva NJ. Squamous cell carcinoma subverts adjacent histologically normal epithelium to promote lateral invasion. J Exp Med. 2021;218(6). 10.1084/jem.20200944. PubMed PMID: 33835136; PMCID: PMC8042603. 10.1084/jem.20200944 PMC8042603 33835136

[27] End C, Bikker F, Renner M, Bergmann G, Lyer S, Blaich S, et al. DMBT1 functions as pattern-recognition molecule for poly-sulfated and poly-phosphorylated ligands. Eur J Immunol. 2009;39(3):833–42. 10.1002/eji.200838689. 19189310 10.1002/eji.200838689

[28] Muller H, End C, Weiss C, Renner M, Bhandiwad A, Helmke BM, et al. Respiratory deleted in malignant brain tumours 1 (DMBT1) levels increase during lung maturation and infection. Clin Exp Immunol. 2008;151(1):123–9. 10.1111/j.1365-2249.2007.03528.x. 17991292 10.1111/j.1365-2249.2007.03528.x PMC2276934

[29] Mollenhauer J, Herbertz S, Holmskov U, Tolnay M, Krebs I, Merlo A, et al. DMBT1 encodes a protein involved in the immune defense and in epithelial differentiation and is highly unstable in cancer. Cancer Res. 2000;60(6):1704–10 (PubMed PMID: 10749143). 10749143

[30] Gopalakrishnan V, Spencer CN, Nezi L, Reuben A, Andrews MC, Karpinets TV, et al. Gut microbiome modulates response to anti-PD-1 immunotherapy in melanoma patients. Science. 2018;359(6371):97–103. 10.1126/science.aan4236. 29097493 10.1126/science.aan4236 PMC5827966

[31] Nagao-Kitamoto H, Kitamoto S, Kamada N. Inflammatory bowel disease and carcinogenesis. Cancer Metastasis Rev. 2022;41(2):301–16. 10.1007/s10555-022-10028-4. 35416564 10.1007/s10555-022-10028-4

[32] Routy B, Le Chatelier E, Derosa L, Duong CPM, Alou MT, Daillere R, et al. Gut microbiome influences efficacy of PD-1-based immunotherapy against epithelial tumors. Science. 2018;359(6371):91–7. 10.1126/science.aan3706. 29097494 10.1126/science.aan3706

[33] Caruso R, Ono M, Bunker ME, Nunez G, Inohara N. Dynamic and asymmetric changes of the microbial communities after cohousing in laboratory mice. Cell Rep. 2019;27(11):3401-12 e3. 10.1016/j.celrep.2019.05.042. 31189120 10.1016/j.celrep.2019.05.042 PMC6690056

[34] Ubeda C, Lipuma L, Gobourne A, Viale A, Leiner I, Equinda M, et al. Familial transmission rather than defective innate immunity shapes the distinct intestinal microbiota of TLR-deficient mice. J Exp Med. 2012;209(8):1445–56. 10.1084/jem.20120504. 22826298 10.1084/jem.20120504 PMC3409501

[35] Wang Z, Wu VH, Allevato MM, Gilardi M, He Y, Luis Callejas-Valera J, et al. Syngeneic animal models of tobacco-associated oral cancer reveal the activity of in situ anti-CTLA-4. Nat Commun. 2019;10(1):5546. 10.1038/s41467-019-13471-0. 31804466 10.1038/s41467-019-13471-0 PMC6895221

[36] Elseragy A, Bello IO, Wahab A, Coletta RD, Makitie AA, Leivo I, et al. Emerging histopathologic markers in early-stage oral tongue cancer: a systematic review and meta-analysis. Head Neck. 2022;44(6):1481–91. 10.1002/hed.27022. 35229398 10.1002/hed.27022 PMC9545479

[37] Brandwein-Gensler M, Smith RV, Wang B, Penner C, Theilken A, Broughel D, et al. Validation of the histologic risk model in a new cohort of patients with head and neck squamous cell carcinoma. Am J Surg Pathol. 2010;34(5):676–88. 10.1097/PAS.0b013e3181d95c37. 20414102 10.1097/PAS.0b013e3181d95c37

[38] Hawkins BL, Heniford BW, Ackermann DM, Leonberger M, Martinez SA, Hendler FJ. 4NQO carcinogenesis: a mouse model of oral cavity squamous cell carcinoma. Head Neck. 1994;16(5):424–32. 10.1002/hed.2880160506. 7960739 10.1002/hed.2880160506

[39] Tang XH, Knudsen B, Bemis D, Tickoo S, Gudas LJ. Oral cavity and esophageal carcinogenesis modeled in carcinogen-treated mice. Clin Cancer Res. 2004;10(1 Pt 1):301–13. 10.1158/1078-0432.ccr-0999-3. 14734483 10.1158/1078-0432.ccr-0999-3

[40] Sagheer SH, Whitaker-Menezes D, Han JYS, Curry JM, Martinez-Outschoorn U, Philp NJ. 4NQO induced carcinogenesis: a mouse model for oral squamous cell carcinoma. Methods Cell Biol. 2021;163:93–111. 10.1016/bs.mcb.2021.01.001. 33785171 10.1016/bs.mcb.2021.01.001

[41] Perez-Pacheco C, Schmitd LB, Furgal A, Bellile EL, Liu M, Fattah A, et al. Increased nerve density adversely affects outcome in oral cancer. Clin Cancer Res. 2023;29(13):2501–12. 10.1158/1078-0432.CCR-22-3496. 37039710 10.1158/1078-0432.CCR-22-3496 PMC10371054

[42] Baker JM, Hinkle KJ, McDonald RA, Brown CA, Falkowski NR, Huffnagle GB, et al. Whole lung tissue is the preferred sampling method for amplicon-based characterization of murine lung microbiota. Microbiome. 2021;9(1):99. 10.1186/s40168-021-01055-4. 33952355 10.1186/s40168-021-01055-4 PMC8101028

[43] Caporaso JG, Lauber CL, Walters WA, Berg-Lyons D, Lozupone CA, Turnbaugh PJ, et al. Global patterns of 16S rRNA diversity at a depth of millions of sequences per sample. Proc Natl Acad Sci U S A. 2011;108(1(Suppl 1)):4516–22. 10.1073/pnas.1000080107. 20534432 10.1073/pnas.1000080107 PMC3063599

[44] Kozich JJ, Westcott SL, Baxter NT, Highlander SK, Schloss PD. Development of a dual-index sequencing strategy and curation pipeline for analyzing amplicon sequence data on the MiSeq Illumina sequencing platform. Appl Environ Microbiol. 2013;79(17):5112–20. 10.1128/AEM.01043-13. 23793624 10.1128/AEM.01043-13 PMC3753973

[45] Schloss PD. A high-throughput DNA sequence aligner for microbial ecology studies. PLoS One. 2009;4(12):e8230. 10.1371/journal.pone.0008230. 20011594 10.1371/journal.pone.0008230 PMC2788221

[46] Wang Q, Cole JR. Updated RDP taxonomy and RDP classifier for more accurate taxonomic classification. Microbiol Resour Announc. 2024;13(4):e0106323. 10.1128/mra.01063-23. 38436268 10.1128/mra.01063-23 PMC11008197

[47] Yoon G, Gaynanova I, Muller CL. Microbial networks in SPRING - semi-parametric rank-based correlation and partial correlation estimation for quantitative microbiome data. Front Genet. 2019;10:516. 10.3389/fgene.2019.00516. 31244881 10.3389/fgene.2019.00516 PMC6563871

[48] Liu H, Roeder K, Wasserman L. Stability Approach to Regularization Selection (StARS) for high dimensional graphical models. Adv Neural Inf Process Syst. 2010;24(2):1432–40. PubMed PMID: 25152607; PMCID: PMC4138724. PMC4138724 25152607

[49] Aitchison J. The statistical analysis of compositional data. J Roy Stat Soc: Ser B (Methodol). 1982;44(2):21. 10.1111/j.2517-6161.1982.tb01195.x.

[50] Goldsmith J, Greven S, Crainiceanu C. Corrected confidence bands for functional data using principal components. Biometrics. 2013;69(1):41–51. 10.1111/j.1541-0420.2012.01808.x. 23003003 10.1111/j.1541-0420.2012.01808.x PMC3962763

[51] Friedman J, Hastie T, Tibshirani R. Regularization paths for generalized linear models via coordinate descent. J Stat Softw. 2010;33(1):1–22. PubMed PMID: 20808728; PMCID: PMC2929880. PMC2929880 20808728

[52] Irfan M, Delgado RZR, Frias-Lopez J. The oral microbiome and cancer. Front Immunol. 2020;11:591088. 10.3389/fimmu.2020.591088. 33193429 10.3389/fimmu.2020.591088 PMC7645040

[53] Farquhar DR, Divaris K, Mazul AL, Weissler MC, Zevallos JP, Olshan AF. Poor oral health affects survival in head and neck cancer. Oral Oncol. 2017;73:111–7. 10.1016/j.oraloncology.2017.08.009. 28939062 10.1016/j.oraloncology.2017.08.009 PMC5659716

[54] Tezal M, Sullivan MA, Hyland A, Marshall JR, Stoler D, Reid ME, et al. Chronic periodontitis and the incidence of head and neck squamous cell carcinoma. Cancer Epidemiol Biomarkers Prev. 2009;18(9):2406–12. 10.1158/1055-9965.EPI-09-0334. 19745222 10.1158/1055-9965.EPI-09-0334

[55] Gong HL, Shi Y, Zhou L, Wu CP, Cao PY, Tao L, et al. The composition of microbiome in larynx and the throat biodiversity between laryngeal squamous cell carcinoma patients and control population. PLoS One. 2013;8(6):e66476. 10.1371/journal.pone.0066476. 23824228 10.1371/journal.pone.0066476 PMC3688906

[56] Goodman B, Gardner H. The microbiome and cancer. J Pathol. 2018;244(5):667–76. 10.1002/path.5047. 29377130 10.1002/path.5047

[57] Faust K, Raes J. Microbial interactions: from networks to models. Nat Rev Microbiol. 2012;10(8):538–50. 10.1038/nrmicro2832. 22796884 10.1038/nrmicro2832

[58] David LA, Maurice CF, Carmody RN, Gootenberg DB, Button JE, Wolfe BE, et al. Diet rapidly and reproducibly alters the human gut microbiome. Nature. 2014;505(7484):559–63. 10.1038/nature12820. 24336217 10.1038/nature12820 PMC3957428

[59] Caporaso JG, Lauber CL, Costello EK, Berg-Lyons D, Gonzalez A, Stombaugh J, et al. Moving pictures of the human microbiome. Genome Biol. 2011;12(5):R50. 10.1186/gb-2011-12-5-r50. 21624126 10.1186/gb-2011-12-5-r50 PMC3271711

[60] Lozupone CA, Stombaugh JI, Gordon JI, Jansson JK, Knight R. Diversity, stability and resilience of the human gut microbiota. Nature. 2012;489(7415):220–30. 10.1038/nature11550. 22972295 10.1038/nature11550 PMC3577372

[61] Dai D, Yang Y, Yu J, Dang T, Qin W, Teng L, et al. Interactions between gastric microbiota and metabolites in gastric cancer. Cell Death Dis. 2021;12(12):1104. 10.1038/s41419-021-04396-y. 34819503 10.1038/s41419-021-04396-y PMC8613192

[62] Chen Y, Huang Y, Ding X, Yang Z, He L, Ning M, et al. A multi-omics study of familial lung cancer: microbiome and host gene expression patterns. Front Immunol. 2022;13:827953. 10.3389/fimmu.2022.827953. 35479075 10.3389/fimmu.2022.827953 PMC9037597

[63] Yang J, Mu X, Wang Y, Zhu D, Zhang J, Liang C, et al. Dysbiosis of the salivary microbiome is associated with non-smoking female lung cancer and correlated with immunocytochemistry markers. Front Oncol. 2018;8:520. 10.3389/fonc.2018.00520. 30524957 10.3389/fonc.2018.00520 PMC6256243

[64] Gopinath D, Menon RK, Wie CC, Banerjee M, Panda S, Mandal D, et al. Differences in the bacteriome of swab, saliva, and tissue biopsies in oral cancer. Sci Rep. 2021;11(1):1181. 10.1038/s41598-020-80859-0. 33441939 10.1038/s41598-020-80859-0 PMC7806708

[65] Dou Y, Ma C, Wang K, Liu S, Sun J, Tan W, et al. Dysbiotic tumor microbiota associates with head and neck squamous cell carcinoma outcomes. Oral Oncol. 2022;124:105657. 10.1016/j.oraloncology.2021.105657. 34915261 10.1016/j.oraloncology.2021.105657

[66] Feng K, Ren F, Shang Q, Wang X, Wang X. Association between oral microbiome and breast cancer in the east Asian population: a Mendelian randomization and case-control study. Thorac Cancer. 2024;15(12):974–86. 10.1111/1759-7714.15280. 38485288 10.1111/1759-7714.15280 PMC11045337

[67] Herreros-Pomares A, Hervas D, Bagan-Debon L, Jantus-Lewintre E, Gimeno-Cardona C, Bagan J. On the oral microbiome of oral potentially malignant and malignant disorders: dysbiosis, loss of diversity, and pathogens enrichment. Int J Mol Sci. 2023. 10.3390/ijms24043466. 36834903 10.3390/ijms24043466 PMC9961214

[68] Gopalakrishnan V, Helmink BA, Spencer CN, Reuben A, Wargo JA. The influence of the gut microbiome on cancer, immunity, and cancer immunotherapy. Cancer Cell. 2018;33(4):570–80. 10.1016/j.ccell.2018.03.015. 29634945 10.1016/j.ccell.2018.03.015 PMC6529202

[69] Hanahan D. Hallmarks of cancer: new dimensions. Cancer Discov. 2022;12(1):31–46. 10.1158/2159-8290.CD-21-1059. 35022204 10.1158/2159-8290.CD-21-1059

[70] Sepich-Poore GD, Zitvogel L, Straussman R, Hasty J, Wargo JA, Knight R. The microbiome and human cancer. Science. 2021. 10.1126/science.abc4552. 33766858 10.1126/science.abc4552 PMC8767999

[71] Teles FRF, Alawi F, Castilho RM, Wang Y. Association or causation? Exploring the oral microbiome and cancer links. J Dent Res. 2020;99(13):1411–24. 10.1177/0022034520945242. 32811287 10.1177/0022034520945242 PMC7684840

[72] Ericson T, Rundegren J. Characterization of a salivary agglutinin reacting with a serotype c strain of Streptococcus mutans. Eur J Biochem. 1983;133(2):255–61. 10.1111/j.1432-1033.1983.tb07456.x. 6852037 10.1111/j.1432-1033.1983.tb07456.x

[73] Holmskov U, Lawson P, Teisner B, Tornoe I, Willis AC, Morgan C, et al. Isolation and characterization of a new member of the scavenger receptor superfamily, glycoprotein-340 (gp-340), as a lung surfactant protein-D binding molecule. J Biol Chem. 1997;272(21):13743–9. 10.1074/jbc.272.21.13743. 9153228 10.1074/jbc.272.21.13743

[74] Mollenhauer J, Wiemann S, Scheurlen W, Korn B, Hayashi Y, Wilgenbus KK, et al. DMBT1, a new member of the SRCR superfamily, on chromosome 10q25.3-26.1 is deleted in malignant brain tumours. Nat Genet. 1997;17(1):32–9. 10.1038/ng0997-32. 9288095 10.1038/ng0997-32

[75] Holmskov U, Mollenhauer J, Madsen J, Vitved L, Gronlund J, Tornoe I, et al. Cloning of gp-340, a putative opsonin receptor for lung surfactant protein D. Proc Natl Acad Sci U S A. 1999;96(19):10794–9. 10.1073/pnas.96.19.10794. 10485905 10.1073/pnas.96.19.10794 PMC17962

[76] Mollenhauer J, Holmskov U, Wiemann S, Krebs I, Herbertz S, Madsen J, et al. The genomic structure of the DMBT1 gene: evidence for a region with susceptibility to genomic instability. Oncogene. 1999;18(46):6233–40. 10.1038/sj.onc.1203071. 10597221 10.1038/sj.onc.1203071

[77] Bikker FJ, Ligtenberg AJ, End C, Renner M, Blaich S, Lyer S, et al. Bacteria binding by DMBT1/SAG/gp-340 is confined to the VEVLXXXXW motif in its scavenger receptor cysteine-rich domains. J Biol Chem. 2004;279(46):47699–703. 10.1074/jbc.M406095200. 15355985 10.1074/jbc.M406095200

[78] Bikker FJ, Ligtenberg AJ, Nazmi K, Veerman EC, van’t Hof W, Bolscher JG, et al. Identification of the bacteria-binding peptide domain on salivary agglutinin (gp-340/DMBT1), a member of the scavenger receptor cysteine-rich superfamily. J Biol Chem. 2002;277(35):32109–15. 10.1074/jbc.M203788200. 12050164 10.1074/jbc.M203788200

[79] Leito JT, Ligtenberg AJ, Nazmi K, de Blieck-Hogervorst JM, Veerman EC, Nieuw Amerongen AV. A common binding motif for various bacteria of the bacteria-binding peptide SRCRP2 of DMBT1/gp-340/salivary agglutinin. Biol Chem. 2008;389(9):1193–200. 10.1515/BC.2008.135. 18713006 10.1515/BC.2008.135

[80] Li J, Metruccio MME, Evans DJ, Fleiszig SMJ. Mucosal fluid glycoprotein DMBT1 suppresses twitching motility and virulence of the opportunistic pathogen Pseudomonas aeruginosa. PLoS Pathog. 2017;13(5):e1006392. 10.1371/journal.ppat.1006392. 28489917 10.1371/journal.ppat.1006392 PMC5440049

[81] Danella EB, de Costa Meiros M, D’Silva NJ. Cytokines secreted by inflamed oral mucosa: implications for oral cancer progression. Oncogene. 2023;42(15):1159–65. 10.1038/s41388-023-02649-y. 36879116 10.1038/s41388-023-02649-y

[82] Bostanci N, Bao K, Wahlander A, Grossmann J, Thurnheer T, Belibasakis GN. Secretome of gingival epithelium in response to subgingival biofilms. Mol Oral Microbiol. 2015;30(4):323–35. 10.1111/omi.12096. 25787257 10.1111/omi.12096

[83] Brown JL, Johnston W, Delaney C, Rajendran R, Butcher J, Khan S, et al. Biofilm-stimulated epithelium modulates the inflammatory responses in co-cultured immune cells. Sci Rep. 2019;9(1):15779. 10.1038/s41598-019-52115-7. 31673005 10.1038/s41598-019-52115-7 PMC6823452

[84] Ebersole JL, Peyyala R, Gonzalez OA. Biofilm-induced profiles of immune response gene expression by oral epithelial cells. Mol Oral Microbiol. 2019. 10.1111/omi.12251. 30407731 10.1111/omi.12251 PMC6335182

[85] Ramage G, Lappin DF, Millhouse E, Malcolm J, Jose A, Yang J, et al. The epithelial cell response to health and disease associated oral biofilm models. J Periodontal Res. 2017;52(3):325–33. 10.1111/jre.12395. 27330034 10.1111/jre.12395 PMC5412879

[86] Stathopoulou PG, Benakanakere MR, Galicia JC, Kinane DF. Epithelial cell pro-inflammatory cytokine response differs across dental plaque bacterial species. J Clin Periodontol. 2010;37(1):24–9. 10.1111/j.1600-051X.2009.01505.x. 20096064 10.1111/j.1600-051X.2009.01505.x PMC2900159

