# Supplementary material for: Loss of salivary agglutinin induces changes in the salivary microbiome and accelerates development of oral cancer
Source: Microbiome. 2026 Apr 10;14:151. doi: 10.1186/s40168-026-02337-5 (PMC13188527; doi:10.1186/s40168-026-02337-5)
Supplement: Supplementary file 2 — Supplementary Material 1. [file 40168_2026_2337_MOESM1_ESM.docx]

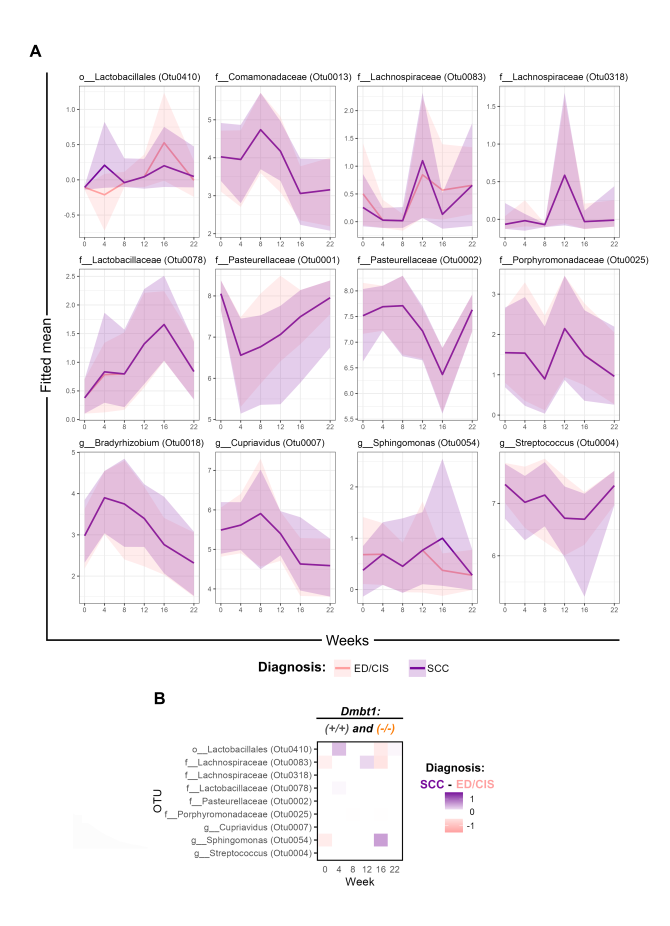


**Figure S1. Significantly different OTUs between diagnosis in *Dmbt1^+/+^* and *Dmbt1^-/-^* mice at any timepoint.** A) Fitted average values (CLR-transformed) for significant OTUs between OSCC and ED/CIS over time in *Dmbt1^+/+^* and *Dmbt1^-/-^* as analyzed by LSVCMM. B) Heatmap summarizing comparisons between *Dmbt1^+/+^* and *Dmbt1^-/-^*.
